# Supplementary material for: Immunonutrition in Acute Geriatric Care: Clinical Outcomes, Inflammatory Profiles, and Immune Responses
Source: Nutrients. 2024 Dec 5;16(23):4211. doi: 10.3390/nu16234211 (PMC11644052; doi:10.3390/nu16234211)
Supplement: Supplementary file 1 [file nutrients-16-04211-s001.zip › nutrients-3326161-supplementary.pdf]

Table S1. Details of antibodies used for flow cytometry analysis.

Name	Clone	Fluorochrome	Manufacturer	Catalogue number
anti-CD19	*HIB19*	PerCP-Cy5.5	ThermoFisher	45–0199
anti-CD3	*OKT3*	APC-eFluor 780	ThermoFisher	47–0037
anti-CD4	*OKT4*	APC	ThermoFisher	17–0048
anti-CD8	*OKT8*	AlexaFluor 700	ThermoFisher	56–0086

Table S2. Primary clinical conditions in absolute value (n) leading to hospitalization.

	CTL	IN	Total
**Delirium**	3	3	6
**Pneumonia**	4	4	8
**Stroke**	0	6	6
**Bowel Obstruction**	2	2	4
**Epilepsy**	2	1	3
**Fever**	2	2	4
**Dehydration**	4	1	5
Total	17	19	36

χ^2= 8.047, p = 0.235.

Table S3. Plasmatic levels of cytokines in the studied population at baseline, stratified by groups of nutrition.

	CTL N = 13	IN N = 10	p
EGF	32.9 ± 20.2	26.7 ± 5.9	.367
Eotaxin	109.6 ± 48.4	86.8 ± 44.3	.260
G-CSF	281.6 ± 83.3	245.6 ± 58.1	.257
GM-CSF	9.1 ± 2.2	9.7 ± 3.2	.590
INF-α2	84.1 ± 16.4	78.8 ± 11.7	.399
INF-γ	21.9 ± 35.2	10.9 ± 4.2	.340
IL-10	17.4 ± 11.8	13.8 ± 6.0	.387
IL-12 p 40	40.95 ± 17.4	39.7 ± 13.2	.857
IL-12 p 70	12.5 ± 15.4	12.5 ± 3.6	.272
IL-13	15.8 ± 25.0	8.2 ± 1.6	.352
IL-15	14.6 ± 6.6	13.7 ± 2.5	.687
IL-17	9.9 ± 10.0	6.5 ± 2.5	.313
IL-1 RA	93.2 ± 41.4	129.8 ± 77.7	.160
IL-1 α	61.5 ± 81.7	38.8 ± 4.7	.393
IL-1 β	3.2 ± 1.5	2.9 ± 1.0	.600
IL-2	6.0 ± 1.8	5.7 ± 1.1	.640
IL-3	1.0 ± 0.18	1.0 ± 0.19	.791
IL-4	93.7 ± 197	42.9 ± 10.4	.397
IL-5	3.1 ± 2.8	1.8 ± 0.4	.173
IL-6	24.7 ± 22.5	17.1 ± 10.4	.336
IL-7	6.7 ± 5.1	6.0 ± 2.1	.712
IL-8	24.5 ± 16.4	18.7 ± 8.2	.316
IP-10	1010.6 ± 448.8	990.2 ± 347.9	.907
MCP-1	503.3 ± 222.7	361.8 ± 171.4	.111
MIP-1 α	15.6 ± 4.5	13.6 ± 1.9	.217
MIP-1 β	90.0 ± 28.8	99.2 ± 52.5	.596

TNF-α	39.2 ± 20.9	38.0 ± 10.9	.870
TNF-β	32.8 ± 43.7	43.7 ± 12.1	.434
VEGF	213.79 ± 79.1	193.8 ± 52.1	.500

EGF (Epidermal Growth Factor); G-CSF (Granulocyte Colony-Stimulating Factor); GM-CSF (Granulocyte-Macrophage Colony-Stimulating Factor; INF-α2 (Interferon-α2); INF-γ (Interferon-γ); IL (Interleukin); MCP-1 (Monocyte Chemoattractant Protein-1); MIP-1 α (Macrophage Inflammatory Protein-1 alpha); MIP-1 β (Macrophage Inflammatory Protein-1 beta); TNF-α (Tumor Necrosis Factor-alpha); TNF-β (Tumor Necrosis Factor-beta); VEGF (Vascular Endothelial Growth Factor).

Table S4. Plasmatic levels of cytokines in the studied population after 1 week, stratified by groups of nutrition.

	**CTL N = 13**	**IN N = 10**	***p***
EGF	35.4 ± 15.1	27.5 ± 11.0	.174
Eotaxin	94.8 ± 56.4	87.7 ± 48.6	.755
G-CSF	265.1 ± 99.9	295.1 ± 69.5	.429
GM-CSF	8.7 ± 1.5	12.6 ± 9.4	.160
INF-A2	78.8 ± 12.8	99.1 ± 28.7	.034
INF- gamma	25.4 ± 43.2	13.9 ± 10.7	.425
IL-10	16.9 ± 8.1	15.8 ± 6.2	.715
IL-12 *p* 40	44.4 ± 18.0	40.6 ± 12.7	.579
IL-12 *p* 70	7.8 ± 6.1	15.7 ± 24.4	.269
IL-13	13.5 ± 16.7	8.3 ± 1.0	.339
IL-15	13.6 ± 5.6	14.9 ± 4.6	.545
IL-17	8.6 ± 5.9	10.3 ± 11.5	.670
IL-1 RA	102.4 ± 90.2	205.7 ± 288.4	.235
IL-1 a	53.4 ±48.1	42.8 ± 8.8	.504
IL-1 b	2.9 ± 1.2	3.7 ± 2.9	.403
IL-2	5.7 ± 1.4	6.3 ± 1.4	.305
IL-3	1.0 ± 0.14	1.1 ± 0.25	.046
IL-4	76.5 ± 115.3	43.5 ± 6.6	.380
IL-5	2.6 ± 1.9	2.4 ± 1.5	.830
IL-6	16.7 ± 9.6	14.9 ± 8.1	.649
IL-7	6.0 ± 3.4	7.1 ± 3.1	.418
IL-8	20.0 ± 10.6	18.4 ± 7.2	.693
IP-10	1017.7 ± 827.8	1439.4 ± 793.0	.231
MCP-1	425.1 ± 229.7	383.6 ± 176.5	.641
MIP-1 a	15.6 ± 4.5	15.0 ± 4.2	.740
MIP-1 b	84.2 ± 28.2	100.2 ± 31.1	.213
TNF-a	39.9 ± 21.5	42.3 ± 8.4	.735
TNF-b	28.1 ± 27.2	23.1 ± 4.8	.576
VEGF	212.6 ± 86.7	216.9 ± 82.4	.904

EGF (Epidermal Growth Factor); G-CSF (Granulocyte Colony-Stimulating Factor); GM-CSF (Granulocyte-Macrophage Colony-Stimulating Factor; INF-α2 (Interferon-α2); INF-γ (Interferon-γ); IL (Interleukin); MCP-1 (Monocyte Chemoattractant Protein-1); MIP-1 α (Macrophage Inflammatory Protein-1 alpha); MIP-1 β (Macrophage Inflammatory Protein-1 beta); TNF-α (Tumor Necrosis Factor-alpha); TNF-β (Tumor Necrosis Factor-beta); VEGF (Vascular Endothelial Growth Factor).

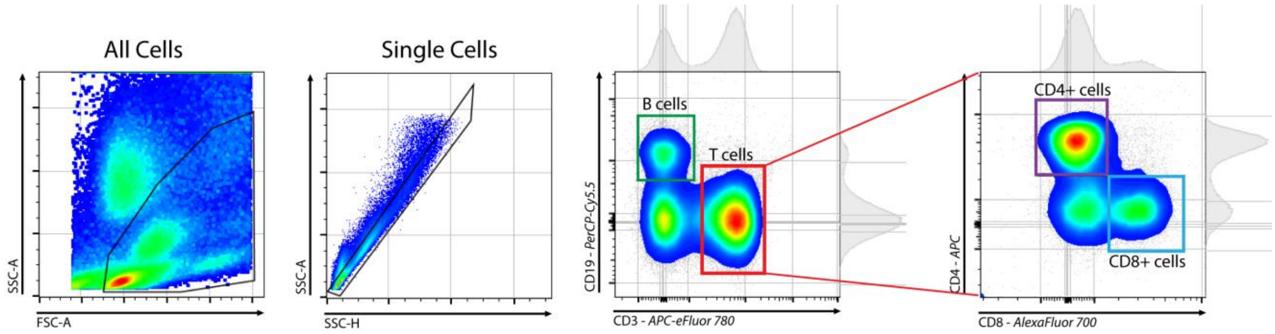

Figure S1. Gating strategy for flow cytometry analysis.
